# Editorial: Case reports in breast cancer 2023-2024

**DOI:** 10.3389/fonc.2025.1711001

**Published:** 2025-11-07

**Authors:** Fengting Yan, Min Sun, Henry Kaplan

**Affiliations:** 1 Swedish Cancer Institute, Seattle, WA, United States; 2 University of Pittsburgh Medical Center, Pittsburgh, PA, United States

**Keywords:** breast cancer, case report, multi omics, DCIS, metastatic breast cancer

Breast cancer remains one of the most prevalent malignancies worldwide, encompassing a wide spectrum of subtypes and therapeutic challenges. While studies utilizing large groups of patients are essential for understanding this disease and its treatment, individual reports of unusual or unrecognized cases can provide valuable insights (1, 2). These unique situations often lead to new research directions and therapies. As the use of multi-omic techniques and the availability of multi-omic data sets become more widespread in this era of precision medicine we will undoubtedly also see reports of important new and hopefully targetable biologic processes through the report of “n of 1” case studies (3, 4). The case reports noted in this compendium illustrate both the intricacies of managing early-stage and metastatic disease and the opportunities afforded by innovative therapies, unexpected clinical scenarios, and advances in personalized medicine. Collectively, these cases underscore the dynamic nature of breast cancer care and the importance of heightened clinical awareness as well as multidisciplinary collaboration.

A striking example involves bone marrow metastasis with necrosis presenting 11 years after an initial diagnosis of ductal carcinoma *in situ* (DCIS). This delayed recurrence underscores the importance of vigilant, long-term surveillance for patients with high-risk features, such as being under 40 years old, the size of the DCIS, nuclear grade, presence of necrosis, multifocality, surgical margins, and the mode of detection, even among those with seemingly early-stage disease.

Innovative approaches are also reshaping local treatment. One report describes intraoperative radiation therapy (IORT) as an alternative for patients who are unable to tolerate conventional external-beam regimens, particularly for those with difficulty staying still, or with vision impairment that prevent them from traveling to receive post-surgical radiation. This underscores the value of tailoring therapy to individual needs, thereby optimizing both compliance and comfort.

The management of triple-negative breast cancer (TNBC) is particularly challenging for patients who are survivors of B-cell acute lymphoblastic leukemia. Key challenges include cardiac toxicities from cumulative dose of doxorubicin and radiation, the use of immune checkpoint inhibitor after allogeneic stem cell transplantation (allo-SCT), and testing for hereditary cancer in patients with history of allo-SCT. These scenarios highlight the complexities involved managing patients with a secondary primary TNBC following successful treatment for acute leukemia and allo-SCT. They underscore the delicate balance between aggressive treatment and the risk of adverse outcomes, reinforcing the importance of individualized strategies guided by multidisciplinary expertise.

Equally significant are the dynamic shifts in tumor biology. Hormone receptor conversion—cases transitioning from negative to positive status, or vice versa—reflects the evolving nature of metastatic disease and the need for periodic reassessment. Additionally, findings from the Plus-ENDO study support the use of ultrasound-based evaluations in advanced hormone receptor–positive disease for patients treated with CDK4/6 inhibitor-based therapy. Ultrasound demonstrated changes in lesions not only in size but also in echogenicity and the absence of vascularization. Ultrasound remains a practical and accessible tool for monitoring local response to medical treatments, particularly in unfit and elderly patients, allowing for a delay in more demanding and expensive exams. This approach has the potential to streamline monitoring and enhance treatment precision.

Rare and atypical presentations continue to challenge conventional diagnostic frameworks. Reports include metaplastic carcinoma, primary breast osteosarcoma, neuroendocrine breast carcinoma with a germline EGFR mutation, and incidental discoveries of invasive lobular carcinoma or anaplastic large cell lymphoma during surgery. Other unusual scenarios—such as ectopic breast cancer in males, renal pelvis metastasis following angiosarcoma surgery, fibroadenoma associated with atypical ductal hyperplasia, and infiltrating epitheliosis mimicking invasive carcinoma—demonstrate the wide range of clinical presentations. Breast cancer presenting as numb cheek syndrome without a discrete mass, as well as breast involvement from extramammary malignancies (including ovarian mucinous carcinoma, rectal carcinoma, papillary thyroid cancer, and endometrial clear cell carcinoma), highlights the need for thorough evaluation to avoid misdiagnosis. In addition, case reports provide valuable insights into the management of rare entities such as giant breast skin warts, malignant phyllodes tumors, and double mammary pseudangiomatous stromal hyperplasia, for which randomized data remain unavailable.

Complex histologies further complicate management. Multi-omic analysis of HER2-enriched, AR-positive breast carcinoma with apocrine differentiation and an oligometastatic course has provided important insights into the genomic and molecular landscape of this rare subtype, underscoring the need for personalized and comprehensive research approaches. Similarly, the report of concurrent breast myeloid sarcoma and a borderline phyllodes tumor illustrates how uncommon combinations can defy standard diagnostic categories, necessitating close multidisciplinary collaboration.

Targeted therapies continue to expand the treatment landscape. The use of Phesgo^®^ (subcutaneous fixed-dose combination of trastuzumab and pertuzumab) in a patient undergoing hemodialysis illustrates the adaptability of HER2-targeted regimens in complex medical contexts. A case involving a young woman who achieved long-term complete remission with a third-line PARP inhibitor following immunotherapy highlights the potential for exceptional responses to targeted therapies. Likewise, novel agents such as fam-trastuzumab deruxtecan, ESG401 (a Trop-2 antibody–drug conjugate), and margetuximab offer new options for HER2-positive metastatic disease. Interestingly, even traditional chemotherapy, such as low-dose continuous 5-FU, has shown the capacity to induce remarkable responses in heavily pretreated TNBC with liver and bone marrow failure.

Emerging therapies, however, also bring new challenges. CDK4/6 inhibitors such as ribociclib, while effective, have been associated with rare toxicities including palinopsia, vitiligo-like reactions, and photosensitivity presenting as dyschromia. PIK3CA inhibitors such as alpelisib have similarly been linked to vitiligo-like toxicity, while urothelial injury has been reported as a rare complication of paclitaxel and trastuzumab. Case experience with ribociclib in a patient with acute hepatitis—an often excluded population—provides additional insight into managing complex comorbidities. These examples highlight the importance of balancing efficacy with safety, careful monitoring, and patient education.

Beyond oncologic control, psychosocial and functional considerations are equally critical. Rehabilitation strategies for lymphedema, for example, can significantly improve quality of life, reinforcing the importance of holistic care that addresses both physical and emotional well-being.

Taken together, these cases reflect the complexity of breast cancer management across the spectrum of disease. They reinforce the central role of personalized medicine, the necessity of integrating supportive care, and the value of sustained vigilance in follow-up. As new therapies expand possibilities and tumor biology continues to reveal its adaptive nature, clinicians must remain both innovative and flexible in their approach.

In conclusion, breast cancer care is defined by complexity, diversity, and continual evolution. The cases highlighted here demonstrate not only the challenges faced by patients and clinicians but also the opportunities created by advances in diagnostics, therapeutics, and multidisciplinary care. Moving forward, the integration of novel treatments with compassionate, individualized strategies will be essential to improving outcomes. With ongoing research and collaboration, we can continue to navigate this evolving landscape with both scientific rigor and human resilience.
